# Evaluation of Factors Affecting Mortality in Patients with Idiopathic Pulmonary Fibrosis: A 10-Year Single-Center Experience

**DOI:** 10.3390/diagnostics16010074

**Published:** 2025-12-25

**Authors:** Tugba Onyilmaz, Serap Argun Baris, Bengugul Ozturk, Gozde Oksuzler Kizilbay, Gozde Selvi Guldiken, Hasim Boyaci, Ilknur Basyigit

**Affiliations:** 1 Department of Pulmonary Medicine, Faculty of Medicine, Kocaeli University, 41380 Izmit, Turkey; 2 Department of Pulmonary Medicine, Kızıltepe State Hospital, 47400 Kızıltepe, Turkey; 3 Department of Pulmonary Medicine, Mehmet Akif Ersoy State Hospital, 17100 Canakkale, Turkey

**Keywords:** idiopathic pulmonary fibrosis, mortality, exacerbation, GAP score, comorbidity

## Abstract

**Background/Objectives**: Idiopathic pulmonary fibrosis (IPF) is a chronic, progressive fibrotic interstitial lung disease with high mortality and limited treatment options. Despite recent therapeutic advances, predicting survival remains challenging. Given the challenge of predicting disease progression in IPF, identifying reliable prognostic markers may support individualized treatment strategies, guide follow-up intensity, and improve clinical decision making. This study aimed to evaluate mortality rates and factors associated with poor prognosis in patients with IPF over a 10-year period at a tertiary care center. **Methods**: Medical records of 268 patients diagnosed with IPF between 2015 and 2024 were retrospectively reviewed. Demographic characteristics, comorbidities, radiological findings, pulmonary function test results, frequency of exacerbations and hospitalizations, treatment details, and survival outcomes were analyzed. Univariate and multivariate logistic regression analyses were performed to identify predictors of mortality. **Results**: This study included 268 patients (77.2% male; median age, 72 years). During a median follow-up of 24 months, 44% (*n* = 118) of patients died. Deceased patients were older (*p* < 0.001) and had higher rates of coronary artery disease, pulmonary embolism, pulmonary hypertension, and malignancy (all *p* < 0.05). A definite UIP pattern was more common among deceased patients (71.2% vs. 52.4%, *p* = 0.02). Acute exacerbations (23.3% vs. 8.1%) and hospitalizations (61.9% vs. 23.3%) were significantly more frequent in this group (*p* < 0.001). In multivariate analysis, GAP score (OR 11.68, *p* = 0.001), pulmonary hypertension (OR 15.39, *p* = 0.02), history of exacerbation (OR 56.2, *p* = 0.04), baseline FVC (OR 1.10, *p* = 0.02), mean platelet volume (OR 0.29, *p* = 0.01), and AST level (OR 1.12, *p* = 0.04) were independent predictors of mortality. **Conclusions**: Despite advances in management, IPF continues to carry a high mortality risk. This study represents one of the largest single-center IPF cohorts from our region with long-term real-life follow-up and additionally evaluates laboratory biomarkers such as MPV and AST, which have not been widely investigated as prognostic indicators in IPF. Advanced age, reduced pulmonary function, comorbidities, and acute exacerbations are major prognostic factors. Early recognition and proactive management of these parameters may help improve survival outcomes.

## 1. Introduction

Idiopathic pulmonary fibrosis (IPF) is a chronic, progressive lung disease of unknown etiology that is characterized by fibrosis and is associated with poor prognosis. The disease predominantly affects men aged >60 years, with a mean survival time ranging between 2 and 5 years, comparable to that of several other malignant disorders [1,2]. Although IPF is considered a rare disease, IPF is associated with high mortality. The reported prevalence rates range from 0.33 to 4.51 per 10,000 individuals worldwide, with substantial geographic variability across Europe, North America, and the Asia-Pacific regions [3]. Consistent with these global trends, recent epidemiological data from Türkiye have indicated a rising incidence and prevalence of IPF. National ILD reports estimate the prevalence of IPF to be approximately 6–10 cases per 100,000 adults [4].

The clinical course of IPF is variable. Although some patients remain stable for long periods, others experience rapid deterioration and death. In clinical practice, no parameter that can reliably predict the clinical course has been identified.

Identifying the factors associated with poor prognosis and mortality in IPF is crucial for personalized patient management, monitoring treatment efficacy, and selecting appropriate endpoints for clinical trials. Factors such as advanced age, reduced diffusion capacity (DLCO), decreased forced vital capacity (FVC), smoking history, comorbidities, and acute exacerbations are reportedly associated with mortality [4,5,6]. In recent years, the introduction of these two antifibrotic agents has marked the most significant advancement in IPF therapy. Pirfenidone and nintedanib are antifibrotic agents used in the treatment of IPF that have been shown to slow disease progression, reduce the decline in pulmonary function, and improve survival [7]. IPF, characterized by the progressive loss of pulmonary function over the years, typically follows a clinical course with high mortality. Acute exacerbation of IPF is defined as an acute, unexplained worsening of dyspnea and pulmonary function [8]. Acute exacerbation of IPF is a severe clinical condition that is associated with high morbidity and mortality. The mean survival time following exacerbation has been reported to be approximately 3–4 months [9]. Despite the treatments applied, hospital mortality rates have been reported to range between 50% and 80% [10].

Previous studies have demonstrated that the prevalence of comorbidities among IPF patients is significantly higher than that among the general population, with most patients presenting with two or more concurrent diseases [11,12,13]. The most frequent comorbidities include pulmonary hypertension, combined pulmonary fibrosis and emphysema, venous thromboembolism, lung cancer, gastroesophageal reflux disease, sleep-related disorders, congestive heart failure, coronary artery disease, diabetes mellitus, depression, and anxiety [4]. Among these, malignancy, pulmonary hypertension, coronary artery disease, and venous thromboembolism are most strongly associated with mortality.

Although the aforementioned factors have been identified as potential predictors of mortality and antifibrotic treatments have shown beneficial effects on survival, the inherently heterogeneous course of the disease, characterized by interindividual variability in progression rates and the occurrence of acute exacerbations, makes mortality prediction challenging [14,15].

This study aimed to present 10-year real-life data from patients with IPF who were followed up in a tertiary referral center in Turkiye since antifibrotic therapies became accessible and to determine the mortality rates and factors associated with mortality in this patient population.

## 2. Materials and Methods

### 2.1. Study Population

This study was conducted retrospectively by reviewing the medical records of 268 patients diagnosed with Idiopathic Pulmonary Fibrosis (IPF) between 1 January 2015 and 31 December 2024 at the Department of Pulmonary Medicine, Kocaeli University Faculty of Medicine. The diagnosis was established in accordance with the international diagnostic and treatment guidelines published by the American Thoracic Society (ATS), European Respiratory Society (ERS), Japanese Respiratory Society (JRS), and Latin American Thoracic Association (ALAT) based on high-resolution computed tomography (HRCT) findings, clinical evaluation, pathological assessment when necessary, and multidisciplinary discussion [1]. UIP and probable UIP patterns on HRCT were defined according to the 2018 ATS/ERS/JRS/ALAT criteria, and final diagnostic confirmation was established through a multidisciplinary ILD board review integrating radiologic, clinical, and pathological data [16]. Patients who met the diagnostic criteria were aged ≥18 years; had at least 6 months of follow-up data; and were diagnosed with IPF based on clinical, radiological, and/or pathological findings (radiologically definite or probable UIP pattern and/or histopathologically confirmed) by the multidisciplinary interstitial lung disease (ILD) council were included in the study. Exclusion criteria included other respiratory diseases, such as sarcoidosis, ILD secondary to rheumatologic diseases, hypersensitivity pneumonitis, and drug-induced lung injury. In addition, patients with a history of occupational exposure or radiological findings inconsistent with the UIP pattern were excluded from the study.

This study was approved by the Ethics Committee of Kocaeli University. The requirement for informed consent was waived owing to the retrospective design based on medical records.

### 2.2. Data Collection

Demographic data of the patients (age, sex, smoking history), physical examination findings, comorbid diseases, spirometry values (FVC, FEV_1_, and DLCO at diagnosis and at month 6), radiological findings (definite UIP/probable UIP), treatment information (use of antifibrotic agents, type of drug, and drug-related adverse effects), the number of exacerbations and hospitalizations, use of long-term oxygen therapy (LTOT) at home, GAP score, mortality status, and survival time were recorded. Acute-phase reactants (CRP and erythrocyte sedimentation rate), complete blood count, blood glucose, renal function tests (creatinine, urea, and BUN), liver function tests (AST and ALT), pulmonary function tests (PFTs), carbon monoxide diffusion test (DLCO), and high-resolution computed tomography (HRCT) findings were also reviewed. Before antifibrotic agents became available (pirfenidone in 2016 and nintedanib in 2017), patients were managed with supportive therapies, including systemic corticosteroids when appropriate, long-term oxygen therapy, antireflux medication, and pulmonary rehabilitation, in line with national practice at that time.

Acute exacerbation of IPF was defined according to the 2016 international ATS/ERS criteria as follows:

(1) Acute worsening or new onset of dyspnea within 30 days;
(2) New bilateral ground-glass opacities or consolidation on HRCT superimposed on a background UIP pattern;
(3) Exclusion of alternative causes such as pulmonary infection, heart failure, pulmonary embolism, or drug toxicity [17].

Hospitalization was defined as admission to the hospital due to respiratory deterioration requiring inpatient evaluation or treatment, including hypoxemia; need for oxygen escalation; or management of suspected exacerbation.

### 2.3. Statistical Analysis

Statistical analyses were performed using IBM SPSS Statistics version 25.0 software (SPSS Inc., Chicago, IL, USA). The normality of the data distribution was evaluated using the Kolmogorov–Smirnov test. Numerically distributed variables with normal distribution were expressed as mean ± standard deviation, while non-normally distributed variables were presented as median (min–max). Categorical variables were expressed as frequency (percentage). For comparisons between two groups, Student’s *t*-test was used for normally distributed variables and the Mann–Whitney U test for non-normally distributed variables. For comparisons between more than two groups, one-way analysis of variance (ANOVA) was used for normally distributed variables, and the Kruskal–Wallis test was used for non-normally distributed variables. Associations between categorical variables were evaluated using Pearson’s chi-square test. To identify the factors associated with mortality, univariate analyses were first performed, followed by binary logistic regression analysis including variables found to be significant in univariate analyses. Additionally, survival analyses were conducted using the Kaplan–Meier method, and differences between groups were assessed using the log-rank test. Missing data were handled using a complete-case approach, as no imputation was performed. For survival analyses, patients who were not regularly followed up in our clinic were cross-checked through the National Health Database to determine their vital status. Patients who were alive at the end of the study period were censored at their last documented follow-up date. Statistical significance was set at *p* < 0.05.

## 3. Results

A total of 268 patients (61 females [22.8%] and 207 males [77.2%]) with a median age of 72 years were included in this study. The median follow-up period was 24 months. During the follow-up period, 44% of the patients (*n* = 118) died. A history of smoking was present in 75.2% of patients, and at least one comorbidity was identified in 76.5%. On physical examination, Velcro crackles were detected in 84.2% of patients, and digital clubbing was observed in 32.8%.

Among these patients, 159 (61%) were diagnosed based on a radiological UIP pattern, 15 (5.7%) through biopsy, and 87 (33.3%) with a radiological probable UIP pattern confirmed as IPF by a multidisciplinary council. Antifibrotic therapy was initiated in 186 patients (70.2%) (*n* = 103 (55.4%) with pirfenidone and *n* = 83 (44.6%) with nintedanib). Drug-related side effects were observed in 53 patients (28.3%), with diarrhea being the most common (42.3%). Treatment discontinuation was necessary in 48 patients (25.8%), most commonly because of noncompliance (39.2%) and diarrhea (21.6%). Acute exacerbation was observed in 39 patients (14.8%), hospitalization history in 108 (40.3%), and need for long-term oxygen therapy (LTOT) in 105 patients (39.3%) (Table 1). The median survival time after diagnosis was 29.5 months.

The mortality rate of the study population was 44% (*n* = 118). No significant association was found between sex and mortality; however, the deceased group had a higher median age (73.5 vs. 69 years; *p* < 0.001). Regarding comorbidities, coronary artery disease (*p* = 0.023), pulmonary embolism (*p* = 0.005), pulmonary hypertension (*p* < 0.001), and malignancy (*p* = 0.01) were significantly more frequent among the deceased patients.

Among the patients who died, the radiological pattern of definite UIP was observed significantly more often than the probable UIP pattern (71.2% vs. 52.4%; *p* = 0.02). No significant differences were found between the groups in terms of treatment use, type of antifibrotic agent, adverse effects, treatment discontinuation, or vaccination status (Table 2).

The frequency of exacerbations (23.3% vs. 8.1%; *p* = 0.001) and hospitalization rate (61.9% vs. 23.3%; *p* < 0.001) were significantly higher in the deceased patients than in the survivors. Among pulmonary function test parameters, baseline FVC and FEV_1_ (in mL and % predicted), 6th-month FVC % predicted, and baseline and 6th-month DLCO % predicted values were significantly lower in the deceased group (*p* < 0.05) (Table 3). Laboratory findings showed that RDW, neutrophil-to-lymphocyte ratio (NLR), aspartate aminotransferase (AST), lactate dehydrogenase (LDH), and neutrophil counts were significantly higher in the deceased patients (*p* < 0.005). Conversely, MPV and albumin and lymphocyte levels were significantly lower in deceased patients than in survivors (*p* < 0.05) (Table 4).

**Figure 1:**
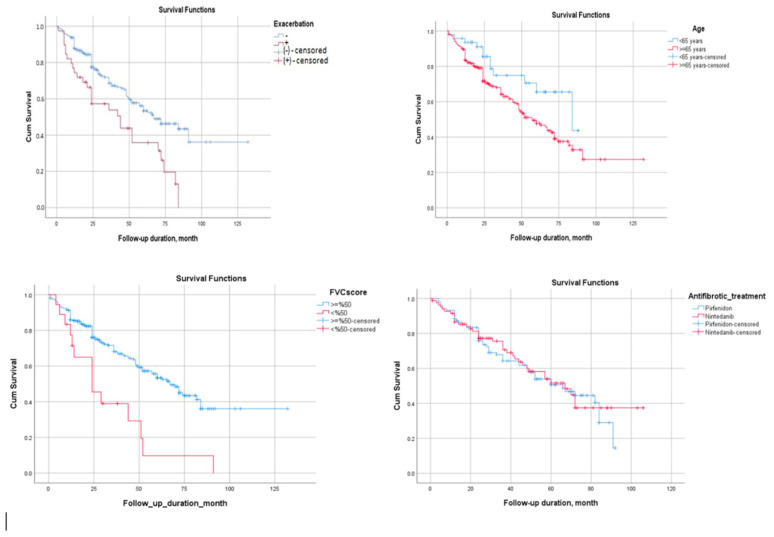
Kaplan–Meier analysis of mortality according to the presence of acute exacerbation, age, FVC value, and type of antifibrotic agent used.

In the binary logistic regression analysis, the following variables were identified as independent risk factors associated with mortality in patients with IPF: GAP score (OR 11.68, 95% CI: 2.63–51.88, *p* = 0.001), presence of pulmonary hypertension (OR 15.39, 95% CI: 1.54–153.67, *p* = 0.02), history of acute exacerbation (OR 56.2, 95% CI: 1.22–2585.8, *p* = 0.039), baseline FVC value (OR 1.103, 95% CI: 1.014–1.2, *p* = 0.022), mean platelet volume (MPV) (OR 0.29, 95% CI: 0.11–0.74, *p* = 0.01), and AST (OR 1.124, 95% CI: 1.005–1.26, *p* = 0.04) (Table 5). In the Kaplan–Meier survival analysis, the presence of acute exacerbation (log-rank test: χ^2^ = 11.12; *p* = 0.001), baseline FVC < 50% (log-rank test: χ^2^ = 12.92; *p*= 0.000), and age ≥ 65 years (log-rank test: χ^2^ = 4.63; *p* = 0.03) were significantly associated with poor prognosis (Figure 1). The survival outcomes of patients receiving pirfenidone and nintedanib were also evaluated using Kaplan–Meier analysis. The survival curves for both antifibrotic agents showed a similar pattern, and no statistically significant difference was found between the two treatment groups (log-rank test: χ^2^ = 0.082; *p* = 0.774) (Figure 1).

## 4. Discussion

In this study, 10-year follow-up data of 268 patients diagnosed with IPF were retrospectively analyzed, and factors associated with mortality were comprehensively evaluated. Our findings revealed that mortality rates in IPF remain high (44%), and that the GAP score, presence of pulmonary hypertension, history of acute exacerbation, low baseline FVC value, and decreased MPV level were independent predictors of mortality.

In our study, the mean survival time from diagnosis was 29.5 months, and the 44% mortality rate was consistent with previously reported 3–5-year survival durations [18,19,20]. Survival is even shorter in elderly patients. Indeed, the significantly higher diagnostic age among the deceased patients in our cohort supports this finding.

In various studies, short-term mortality has been associated with older age, male sex, low body mass index (BMI), reduced or progressively declining pulmonary function, use of oxygen therapy, and hospitalization due to respiratory causes [21,22,23]. In our study, no relationship was found between sex and mortality; however, the patients in the mortality group were older. Additionally, although there was no statistically significant relationship between BMI and mortality, long-term oxygen therapy (LTOT) was associated with higher mortality. Among pulmonary function parameters, baseline FVC and FEV_1_ (mL and % predicted), 6th-month FVC % predicted, and baseline and 6th-month DLCO % predicted values were lower in deceased patients compared to survivors.

Although death in IPF most commonly occurs due to disease progression or causes, such as pneumonia, comorbidities can also play a significant role in mortality. Recent management strategies emphasize that optimal care in IPF extends beyond antifibrotic therapy and requires systematic identification and treatment of comorbidities, including lung cancer, pulmonary hypertension, cardiovascular disease, and thromboembolic events, which substantially influence survival [3]. In particular, cardiovascular diseases and lung cancer have been highlighted in various studies for their impact on mortality [13]. In the study conducted by Song et al., the median age and sex distribution of patients with IPF were similar to those in our cohort, while the prevalence of lung cancer was slightly lower at 6.4% compared with 9.5% in our study. Consistent with our findings, the mortality rate was higher among patients with IPF diagnosed with lung cancer, and the presence of lung cancer was identified as an independent risk factor for mortality [24]. In a study conducted by Erten et al., mortality was significantly higher in patients with chronic renal failure [25]. Two studies also reported an association between pulmonary hypertension and lung cancer with mortality in patients with IPF [26,27]. In our study, among the comorbidities, coronary artery disease, pulmonary embolism, pulmonary hypertension, and malignancy were more frequent in the patients who died. Furthermore, the presence of pulmonary hypertension was identified as an independent risk factor for mortality. Pulmonary hypertension (PH) was a strong independent predictor of mortality in our cohort, consistent with previous reports indicating that PH contributes to accelerated clinical decline in IPF. PH increases right ventricular afterload and accelerates right-heart failure, leading to substantially higher mortality in IPF [28]. In addition, echocardiographic monitoring enables early detection of rising pulmonary pressures, supporting timely intervention and optimized management [11]. Taken together, these findings reinforce the need for routine echocardiographic monitoring and proactive management strategies in patients at risk of or exhibiting signs of PH, and further suggest that comorbidities may play a decisive role in the prognosis of IPF.

Our findings underscore the importance of integrated, multidisciplinary care in IPF, particularly given the contributions of pulmonary hypertension, cardiac involvement, and liver-related biomarkers such as AST to the overall prognosis. Coordinated surveillance involving pulmonology, cardiology, and hepatology may facilitate the early detection of systemic complications and allow for more proactive management. Such an approach may ultimately improve risk stratification and patient outcomes.

In our cohort, the GAP score emerged as one of the strongest independent predictors of mortality, reinforcing its value as a practical and reliable risk-stratification tool in real-life clinical settings. Clinically, this finding underscores the importance of routinely incorporating GAP scoring into patient assessment to guide follow-up intensity, anticipate clinical deterioration, and tailor antifibrotic therapy decisions. Previous studies have similarly highlighted the prognostic utility of the GAP index [29,30], and a meta-analysis evaluating mortality-related risk factors in IPF demonstrated that the GAP index is an independent predictor of mortality in patients with IPF [31]. Similarly, Tsubouchi et al. reported that a lower FVC% predicted—a key component of the GAP score—was independently associated with IPF-related mortality [32]. The ability of the GAP score to consistently predict outcomes across different healthcare systems and demographic profiles strengthens its role as a cornerstone parameter for prognostic evaluation in IPF, and an accurate assessment of this index may facilitate timely interventions aimed at improving patient outcomes. Our findings suggest that the prognostic assessment of IPF may be improved by integrating multiple dimensions of risk. In particular, composite models that combine the GAP score with laboratory biomarkers such as MPV and structured comorbidity indices could provide more nuanced, individualized prognostic stratification. Future studies should aim to develop and externally validate such multidimensional risk scores to better guide clinical decision-making.

A history of acute exacerbation negatively affects the survival of IPF patients. The management of acute exacerbations of idiopathic pulmonary fibrosis remains largely supportive, as no therapy has been conclusively shown to reverse acute lung injury or significantly improve survival. High-dose systemic corticosteroids and broad-spectrum antibiotics are commonly administered in clinical practice, despite limited evidence from randomized controlled trials, given the difficulty in excluding infectious triggers at presentation. Supportive care, including oxygen supplementation and noninvasive or invasive ventilatory support when indicated, is the cornerstone of treatment for AE-IPF [3]. In a retrospective study by Song et al., which included 461 patients with IPF, acute exacerbation occurred in 96 patients (20.8%), and the in-hospital mortality rate in this group was reported to be 50%, while the early mortality rate was 60% [33]. Similarly, in another study analyzing 598 patients with IPF, acute exacerbation was observed in 58 patients (9.8%), with in-hospital and 3-month mortality rates of 56.9% and 63%, respectively [34]. In our study, the frequency of acute exacerbations and hospitalization rates were higher in patients who died than in those who survived. Moreover, exacerbation was identified as an independent risk factor for mortality.

Numerous studies have identified low FVC as an independent predictor of mortality in IPF patients [35,36]. In a meta-analysis conducted by Sun et al., reduced FVC, FVC%, and DLCO% values were associated with a higher risk of mortality in IPF patients [31]. In our study, baseline FVC and FEV_1_ (mL and % predicted), 6th-month FVC% predicted, and baseline and 6th-month DLCO% predicted values were significantly lower in patients who died than in survivors. Furthermore, a lower baseline FVC% was found to be an independent risk factor for mortality. This finding suggests that early diagnosis before a decline in FVC values may help alter the unfavorable course of the disease.

In our study, low MPV and high AST levels were identified as independent risk factors for mortality. Interestingly, in our cohort, lower MPV values were independently associated with higher mortality. Although MPV is typically regarded as a marker of platelet activation, an inverse association may reflect the complex platelet dynamics and microvascular alterations observed in advanced IPF. Progressive fibrosis is characterized by endothelial injury, microvascular rarefaction, and increased platelet consumption, which may result in smaller circulating platelets and lower MPV [37,38]. Additionally, chronic systemic inflammation and advanced disease may impair megakaryocyte maturation, further contributing to a reduced MPV. MPV is an indicator of platelet activation and systemic inflammation and has been associated with vascular injury and poor clinical outcomes [38]. Elevated AST, on the other hand, is considered a biomarker that reflects not only hepatic injury but also systemic stress, muscle damage, and the inflammatory process [39]. In a study evaluating 59 patients who required intensive care unit treatment for acute exacerbation of IPF between 2017 and 2023, MPV and MPV/platelet ratio values were assessed, and the median MPV of non-survivors was found to be higher than that of survivors; however, this difference did not reach statistical significance [40]. Consistent with these findings, an observational study in patients with pulmonary arterial hypertension (PAH) demonstrated that MPV was significantly higher in PAH patients than in healthy controls but was not predictive of prognosis [36], while another study showed that increased MPV has prognostic significance in acute myocardial infarction [38]. Taken together, these observations suggest that MPV and AST may reflect broader vascular and inflammatory pathways contributing to disease severity, underscoring their potential value as complementary prognostic markers in IPF. Therefore, further research is needed to elucidate the potential impact of MPV and AST level on mortality risk in patients with IPF.

Recent studies suggest that longitudinal serum biomarkers such as KL-6, surfactant protein-D (SP-D), etc., may refine prognostic assessment in fibrotic ILD and IPF. Higher baseline levels and serial increases in KL-6 and SP-D have been associated with greater fibrotic extent, accelerated functional decline, and poorer survival in IPF, particularly when measured during antifibrotic therapy [41]. However, biomarkers such as KL-6, SP-D, and ferritin are not routinely measured in our clinical practice, and therefore these longitudinal data were not available in this retrospective study. Additionally, CRP levels were not assessed in our analysis. Prospective studies incorporating these biomarkers are needed to better elucidate their potential prognostic value in IPF. Because numerous molecular pathways contribute to fibrogenesis, it is recommended that future treatments adopt a multi-target strategy and incorporate multiple biomarkers [3].

In a study investigating mortality predictors in 119 patients with IPF receiving antifibrotic therapy, elevated RDW and NLR levels were identified as predictors of mortality. Similarly, in our study, although RDW and NLR values were significantly higher in non-survivors than in survivors, they were not identified as significant factors in the binary logistic regression analysis [42].

In our study, a definite UIP pattern was observed significantly more frequently among deceased patients than among those with a probable UIP pattern, which is consistent with the literature. Gayá et al. reported that patients with a probable UIP pattern had a 30% higher survival rate than those with a definite UIP pattern [43]. Similarly, Chen et al. demonstrated that IPF patients with a definite UIP pattern had higher mortality than those with a probable UIP pattern [44].

Currently, two antifibrotic agents (pirfenidone and nintedanib) are recommended for IPF treatment. Although these therapies do not provide a cure, they aim to slow the clinical and functional deterioration. Currently, antifibrotic therapy is the only treatment shown to improve survival in IPF [4]. However, similarly to our study, several reports have found no significant difference in survival between patients receiving antifibrotic therapy and those who did not. In a study by Erten et al., there was no significant difference in mortality between patients who received antifibrotic therapy and those who did not [25]. Similarly, another study reported no difference in survival between patients with IPF treated with pirfenidone and those treated with Nintedanib [45]. A retrospective analysis conducted between 2011 and 2019, including 263 IPF patients, also demonstrated no survival difference between the groups treated with pirfenidone and nintedanib [46]. In our study, it was suggested that the group of patients not receiving antifibrotic therapy may consist of individuals with severe respiratory impairment, those who did not tolerate therapy, or those with very mild disease who refused treatment, potentially biasing the results. Given the favorable survival outcomes associated with antifibrotic therapy, it should be recommended for all eligible patients, and careful management of adverse effects is crucial to ensure adherence to treatment.

Future research should aim to validate these findings in prospective cohorts and incorporate advanced computational tools to refine prognostic accuracy. Machine-learning–based models that integrate clinical variables, HRCT features, and laboratory biomarkers hold significant potential for improving individualized risk prediction in IPF. Such approaches may ultimately support more precise patient stratification and guide development of targeted management strategies.

This study has several limitations. As our study was a retrospective real-life cohort study, some laboratory variables had missing values. All eligible patients were included regardless of missing laboratory results, and no imputation was performed; instead, a complete-case approach was used for the analysis, which avoided potential bias introduced by imputation but may have reduced statistical power. The retrospective design also introduces the potential for selection bias and incomplete follow-up, as the data availability varied among patients. Additionally, the single-center design may limit the generalizability of the findings. Furthermore, although baseline and 6-month pulmonary function measurements were available, longitudinal lung function decline, biomarker trends, and radiological progression could not be systematically evaluated in all patients because of variability in follow-up intervals, which may limit the interpretation of disease progression patterns. Despite these limitations, the study provides valuable real-life data from a large IPF cohort and offers important insights into mortality-related prognostic factors.

In conclusion, the fact that most prognostic determinants identified in our study are easily accessible in clinical practice indicates that these findings can directly contribute to daily patient management. Despite advancements in diagnosis and treatment, IPF remains a disease with a high mortality rate. In our study, the main factors associated with mortality were advanced age, reduced pulmonary function, acute exacerbations, and comorbidities. Preventing exacerbations, minimizing the loss of lung function, effectively managing comorbidities, and regularly monitoring the GAP score may help reduce mortality. Furthermore, future studies evaluating treatment efficacy should consider these factors.

## 5. Conclusions

This study demonstrated that IPF has a high mortality rate and that numerous clinical, functional, and laboratory parameters significantly influence survival outcomes. The GAP score, presence of pulmonary hypertension, history of acute exacerbation, low baseline FVC, and low MPV levels were identified as independent risk factors associated with mortality in IPF. Our findings emphasize the necessity for early risk stratification and close monitoring of high-risk patient groups in IPF management. Prevention of acute exacerbations, regular follow-up of pulmonary function, management of comorbidities, and evaluation of prognostic biomarkers (such as MPV, AST) may play key roles in individualized treatment strategies. However, the retrospective and single-center design of this study, along with its relatively limited sample size, restrict the generalizability of the results. Future prospective, multicenter, and biomarker-focused studies are needed to validate these findings further and facilitate their integration into clinical practice.

## Figures and Tables

**Table 1:** Demographic and diagnostic characteristics of patients with IPF (*n*: 268).

Demographic **Characteristics**		
Age, years	Median/(25th–75th percentiles)	72 (67–77)
Age of Diagnosis	Median/(25th–75th percentiles)	68 (63–72)
Gender, *n* (%)	Women	61 (22.8%)
	Men	207 (77.2%)
Smoking history, *n* (%)	Non-smoker	66 (24.8%)
	Current smoker	39 (14.7%)
	Former smoker	161 (60.5%)
Smoking pack-years	Median/(25th–75th percentiles)	35 (20–50)
BMI, kg/m^2^	Median/(25th–75th percentiles)	25.8 (23.6–27.5)
Occupation, *n* (%)	Housewife	49 (18.6%)
	Farmer	16 (6.1%)
	Worker	57 (21.6%)
	Officer	19 (7.2%)
	Others	123 (46.5%)
Family history, *n* (%)		8 (3.1%)
Presence of any comorbidity, *n* (%)		202 (76.5%)
	Hypertension	172 (65.6%)
	Coronary artery disease	112 (43.9%)
	Congestive heart failure	40 (15.7%)
	Diabetes mellitus	82 (31.5%)
	Cerebrovascular disease	26 (10.2%)
	Chronic renal disease	39 (15.4%)
	Pulmonary embolism	6 (2.3%)
	Pulmonary hypertension	56 (21.6%)
	Previous malignancy	42 (16%)
	Peptic ulcers/GOR	15 (5.9%)
New-onset lung malignancy		25 (9.5%)
Clinical and diagnostic characteristics		
Initial symptom, *n* (%)	Cough	192 (71.9%)
	Dyspnea	212 (79.1%)
	Sputum	77 (28.7%)
Physical examination	Velcro ral	223 (84.2%)
	Clubbing	86 (32.8%)
Thorax CT, *n* (%)	UIP	161 (60.8%)
	Probable UIP	104 (39.2%)
Emphysema in Thorax CT, *n* (%)		95 (36.4%)
Diagnosis, *n* (%)	Radiologically UIP pattern	159 (61%)
	Probable UIP pattern + MDT-ILD	87 (33.3%)
	Biopsy confirmed UIP	15 (5.7%)
Bronchoalveolar lavage, *n* (%)		35 (13.1%)
CD4/CD8	Median/(25th–75th percentiles)	1.42 (0.8–2.6)
GAP Score	Median/(25th–75th percentiles)	4 (3–5)
Treatment		
Pharmacological treatment, *n* (%)	(+)	186 (70.2%)
	Follow-up without antifibrotic	79 (29.8%)
Antifibrotic medication, *n* (%)	Pirfenidone	103 (55.4%)
	Nintedanib	83 (44.6%)
Adverse effects, *n* (%)		53 (28.3%)
	Nausea/Vomiting	4 (7.7%)
	Diarrhea	22 (42.3%)
	Photosensitivity	7 (13.5%)
	Other	19 (36.5%)
Treatment discontinuation, *n* (%)		48 (25.8%)
Cause of treatment discontinuation, *n* (%)	Abnormal liver function tests	9 (17.6%)
	Diarrhea	11 (21.6%)
	Photosensitivity	5 (9.9%)
	GIS symptoms	2 (3.9%)
	Non-adherence to medication	20 (39.2%)
	Progression of disease	4 (7.8%)
Treatment switch, *n* (%)	None	158 (86.8%)
	Pirfenidone to Nintedanib	14 (7.7%)
	Nintedanib to Pirfenidone	10 (5.5%)
Vaccination—Influenza, *n* (%)		36 (13.4%)
Vaccination—Pneumococcus, *n* (%)		160 (59.7%)
Clinical outcomes		
Follow up duration, month	Median/(25th–75th percentiles)	24 (10–51)
Presence of exacerbation, *n* (%)		39 (14.8%)
Hospitalization, *n* (%)		108 (40.3%)
Total hospitalization, days	Median/(25th–75th percentiles)	11 (6–20)
Need of LTOT, *n* (%)		105 (39.3%)
Overall survival, month	Median/(25th–75th percentiles)	29.5 (18–60)
Exitus, *n* (%)		118 (44%)

Abbreviation: GOR: Gastroozefagial reflux; CT: Computed tomography; UIP: Usual Interstitial Pneumonia; MDT-ILD: Multidisciplinary team-Interstitial lung disease. ‘(+)’ follow-up with antifibrotic.

**Table 2:** Univariate analysis of mortality in patients with IPF—a comparison of demographic, clinic, radiological and diagnostic characteristics, and pulmonary function test results according to the presence of mortality in patients with IPF.

		Mortality		
		(+) *n*: 118	(−) *n*: 150	*p*
Demographic characteristic				
Age, years *		73.5 (69–78)	69 (66–76)	<0.001
Age of Diagnosis *		69 (64–72.3)	67 (62–72)	0.024
Gender, *n* (%)	Women	25 (21.2%)	36 (24%)	0.59
	Men	93 (78.8%)	114 (76%)	
Smoking history, *n* (%)	Non-smoker	31 (26.7%)	35 (23.3%)	
	Current smoker	6 (5.2%)	33 (22%)	0.001
	Former smoker	79 (68.1%)	82 (54.7%)	
Smoking package years *		35 (26.3–50)	35 (20–50)	0.4
BMI, kg/m^2^ *		26 (23.3–28.6)	25.8 (23.4–27.4)	0.9
Family history, *n* (%)		4 (3.4%)	4 (2.8%)	0.7
Presence of any comorbidity, *n* (%)		90 (77.6%)	112 (75.7%)	0.7
	Hypertension	78 (67.2%)	94 (64.4%)	0.63
	Coronary artery disease	59 (51.8%)	53 (37.6%)	0.023
	Congestive heart failure	20 (17.5%)	20 (14.2%)	0.46
	Diabetes mellitus	37 (31.9%)	45 (31%)	0.9
	Cerebrovascular disease	9 (7.9%)	17 (12.2%)	0.3
	Chronic renal disease	17 (14.9%)	22 (15.7%)	0.9
	Pulmonary embolism	6 (5.2%)	0	0.005
	Pulmonary hypertension	37 (32.2%)	19 (13.2%)	<0.001
	Previous malignancy	23 (20%)	19 (12.9%)	0.12
	Peptic ulcers/GOR	6 (5.3%)	9 (6.4%)	0.7
New-onset lung malignancy, *n* (%)		17 (14.5%)	8 (5.5%)	0.01
*Clinical and diagnostic characteristics*				
Initial symptom, *n* (%)	Cough	91 (77.8%)	101 (67.3%)	0.06
	Dyspnea	108 (91.5%)	104 (69.3%)	<0.001
	Sputum	45 (38.1%)	32 (21.3%)	0.003
Physical examination	Velcro ral	104 (88.9%)	119 (80.4%)	0.06
	Clubbing	39 (33.6%)	47 (32.2%)	0.8
Thorax CT, *n* (%)	UIP	84 (71.2%)	77 (52.4%)	0.002
	Probable UIP	34 (28.8%)	70 (47.6%)	
Emphysema in Thorax CT, *n* (%)		41 (34.7%)	54 (37.8%)	0.6
Diagnosis, *n* (%)	Radiologically UIP	84 (71.8%)	75 (52.1%)	
	Probable UIP + MDT-ILD	28 (23.9%)	59 (41%)	0.005
	Biopsy confirmed UIP	5 (4.3%)	10 (6.9%)	
GAP Score *		4 (3–5)	3 (2–4)	<0.001
*Treatment*				
Pharmacological treatment, *n* (%)	(+)	87 (74.4%)	99 (66.9%)	0.19
	Follow-up without antifibrotic	30 (25.6%)	49 (33.1%)	
Antifibrotic medication, *n* (%)	Pirfenidone	51 (58.6%)	52 (52.5%)	0.4
	Nintedanib	36 (41.4%)	47 (47.5%)	
Adverse effects, *n* (%)		21 (24.1%)	32 (32%)	0.23
	Nausea/Vomiting	2 (10.5%)	2 (6.1%)	
	Diarrhea	9 (47.4%)	13 (39.4%)	0.83
	Photosensitivity	2 (10.5%)	5 (15.2%)	
	Others	6 (31.6%)	13 (39.3%)	
Treatment discontinuation, *n* (%)		21 (24.4%)	27 (27%)	0.69
Cause of treatment discontinuation, *n* (%)	Abnormal liver function tests	3 (13%)	6 (21.4%)	
	Diarrhea	6 (26.1%)	5 (17.9%)	0.73
	Photosensitivity	2 (8.7%)	3 (10.7%)	
	GIS symptoms	0	2 (7.1%)	
	Non-adherence to medication	10 (43.5%)	10 (35.8%)	
	Progression of disease	2 (8.7%)	2 (7.1%)	
Treatment switch, *n* (%)	None	71 (84.6%)	87 (88.8%)	
	Pirfernidone to Nintedanib	7 (8.3%)	7 (7.1%)	0.6
	Nintedanib to Pirfenidone	6 (7.1%)	4 (4.1%)	
Vaccination—Influenza, *n* (%)		14 (11.9%)	22 (14.7%)	0.5
Vaccination—Pneumococcus, *n* (%)		63 (53.4%)	97 (64.7%)	0.06
*Clinical outcomes*				
Follow up duration, month *		20 (7–36.5)	27 (12–60)	0.001
Presence of exacerbation, *n* (%)		27 (23.3%)	12 (8.1%)	0.001
Hospitalization, *n* (%)		73 (61.9%)	35 (23.3%)	<0.001
Number of hospitalization *		2 (1–3)	1 (1–2)	0.02
Total hospitalization, days *		14 (8–22)	7 (5–15)	0.001
Need of LTOT, *n* (%)		71 (60.2%)	34 (22.8%)	<0.001
Overall survival, month *		24 (12–48)	43.5 (23–71)	<0.001

Abbreviations: GOR: Gastroozefagial reflux; CT: Computed tomography; UIP: Usual Interstitial Pneumonia; GAP: Gender, age, physiology; MDT-ILD: Multidisciplinary team-Interstitial lung disease; LTOT: Long-term oxygen therapy. * Continuous variables were shown as Median/(25th–75th percentiles). ‘(+)’ deceased patients and ‘(–)’ surviving patients.

**Table 3:** Comparison of pulmonary function test between mortal and survival groups.

	Mortality		
	(+) *n*: 118	(−) *n*: 150	*p*
Initial FVC, mL	2290.8 ± 813.7	2645.8 ± 778	0.013
6th month FVC, mL	2492.5 ± 751.8	2797.5 ± 628.5	0.08
1st year FVC, mL	2550 ± 1006.8	2751.1 ± 716.8	0.46
Last control FVC, mL	2161 ± 1055.7	2647.4 ± 683.7	0.09
Initial FVC, % predicted	67 ± 20.4	77.8 ± 15.6	<0.001
6th month FVC, % predicted	68.9 ± 17.6	79.8 ± 14	0.007
1st year FVC, % predicted	69 ± 23.2	102.5 ± 141.2	0.4
Last control FVC, % predicted	65.9 ± 19.3	76.2 ± 14.2	0.07
Initial FEV_1_, mL	1943.8 ± 609.3	2399 ± 1864.2	0.04
6th month FEV_1_, mL	2135.4 ± 517	2235 ± 474.9	0.42
1st year FEV_1_, mL	2123.8 ± 710.4	2287 ± 610	0.45
Last control FEV_1_, mL	1829 ± 785.7	2140.6 ± 563.1	0.17
Initial FEV_1_, % predicted	72.4 ± 19.2	81.6 ± 17.4	0.002
6th month FEV_1_, % predicted	76.6 ± 16	81.3 ± 14	0.21
1st year FEV_1_, % predicted	71.1 ± 23.4	80.9 ± 15.8	0.11
Last control FEV_1_, % predicted	70.1 ± 23.4	80.6 ± 16.3	0.12
Initial FEV_1_/FVC	86.9 ± 9.7	84.8 ± 11.7	0.3
6th month FEV_1_/FVC	88.6 ± 11.2	80.3 ± 10.9	0.004
1st year FEV_1_/FVC	81.6 ± 8.6	83.4 ± 17	0.7
Last control FEV_1_/FVC	83.9 ± 6	83.2 ± 9.9	0.8
Initial DLCO, % predicted	40.6 ± 15.1	56.2 ± 15.3	<0.001
6th month DLCO, % predicted	38.3 ± 14.5	57.5 ± 15	<0.001
1st year DLCO, % predicted	46.5 ± 19.1	55.9 ± 11.3	0.11
Last control DLCO, % predicted	35.6 ± 17.9	53.6 ± 14.7	0.055

Abbreviations: FEV_1_: Forced Expiratory Volume in 1 s; FVC: Forced Vital Capacity; DLCO: Diffusing Capacity of the Lung for Carbon Monoxide. ‘(+)’ deceased patients and ‘(–)’ surviving patients.

**Table 4:** Comparison of baseline laboratory parameters results between mortal and survival groups.

Mortality			
	(+) *n*: 118	(−) *n*: 150	*p*
WBC, ×10^3^/µL *	9.02 ± 2.3	8.66 ± 2.3	0.21
Neutrophil count, ×10^3^/µL **	5.5 (4.3–6.7)	5.1 (3.8–6.3)	0.02
Neutrophil, % *	62.5 ± 10.8	59.4 ± 10.2	0.015
Lymphocyte count, ×10^3^/µL **	2.1 (1.6–2.7)	2.3 (1.8–3.1)	0.048
Lymphocyte, % *	24.98 ± 9.1	28.2 ± 9.1	0.004
Monosit, % **	8.2 (6.4–9.9)	8.4 (7–9.7)	0.4
Hemoglobin, (g/dL) *	13.6 ±1.6	13.7 ± 1.7	0.7
Eosinofil % **	2.9 (1.7–4.3)	2.6 (1.4–4.1)	0.3
Platelet, ×10^3^/µL **	241.1 (194–281)	236.5 (199–306)	0.3
MPV, (fL) **	8.5 (7.6–9.3)	9.7 (8.6–10.4)	<0.001
RDW-CV, % **	14.8 (13.9–15.6)	13.9 (13.2–15.1)	<0.001
NLR **	2.5 (1.9–3.9)	1.96 (1.5–3.1)	0.002
Urea, (mg/dL) **	34.8 (27.9–42.5)	32.1 (26.1–40.7)	0.2
Total bilirubin, (mg/dL) **	0.5 (0.4–0.7)	0.4 (0.3–0.7)	0.06
Direct bilirubin, (mg/dL) **	0.14 (0.1–0.2)	0.14 (0.1–0.2)	0.5
Albumin, (g/L) **	38.9 (35.8–42)	41.8 (39–44)	<0.001
Total protein, (g/L) **	72.4 (67.7–76)	72.4 (70–75.3)	0.8
AST, (U/L) **	22 (17.9–27.9)	20.4 (16.2–24.7)	0.04
ALT, (U/L) **	15.6 (11.1–22.8)	17 (12.5–23.3)	0.2
LDH, (U/L) **	261.5 (212–320)	221.5 (191–267)	<0.001

Abbreviations: WBC: White Blood Cell count; MPV: Mean Platelet Volume; RDW-CV: Red Cell Distribution Width–Coefficient of Variation; NLR: Neutrophil-to-Lymphocyte Ratio; AST: Aspartate Aminotransferase; ALT: Alanine Aminotransferase; LDH: Lactate Dehydrogenase; fL: Femtoliter; U/L: Units per Liter; mg/dL: Milligrams per Deciliter; g/dL: Grams per Deciliter; g/L: Grams per Liter; ×10^3^/μL: Thousand per microliter. * Mean ± Standard Deviation. ** Median/(25th–75th percentiles). ‘(+)’ deceased patients and ‘(–)’ surviving patients.

**Table 5:** Binary logistic regression analysis; risk factors associated with mortality in patients with IPF.

Variable	OR [95% CI]	*p*
Age	0.81 [0.65–1.01]	0.064
Dyspnea	2.26 [0.14–37.16]	0.57
Sputum	5.82 [0.6–56.56]	0.13
New-onset lung malignancy	2.62 [0.27–25.64]	0.41
Thorax CT (UIP/Probable UIP)	3.13 [0.41–23.76]	0.27
GAP Score	11.68 [2.63–51.88]	0.001
Pulmonary Hypertension	15.39 [1.54–153.67]	0.02
Exacerbation	56.2 [1.22–2585.82]	0.039
LTOT	0.696 [0.09–5.54]	0.73
Initial FVC%predicted	1.103 [1.014–1.2]	0.022
NLR	1.41 [0.7–2.83]	0.34
MPV	0.29 [0.11–0.74]	0.01
RDW	1.064 [0.52–2.18]	0.87
Albumin	1.07 [0.94–1.21]	0.33
AST	1.124 [1.005–1.26]	0.041
LDH	0.99 [0.98–1.006]	0.29

Abbreviations: OR: Odds Ratio; CI: Confidence Interval; GAP: Gender-Age-Physiology; UIP: Usual Interstitial Pneumonia; FVC%: Forced Vital Capacity, percentage of predicted; MPV: Mean Platelet Volume; NLR: Neutrophil-to-Lymphocyte Ratio; RDW: Red Cell Distribution Width; AST: Aspartate Aminotransferase; LDH: Lactate Dehydrogenase; LTOT: Long-Term Oxygen Therapy.

## Data Availability

The data presented in this study are available on request from the corresponding author. The data are not publicly available due to privacy regulations.

## Abbreviations

The following abbreviations are used in this manuscript:

ALAT	Latin American Thoracic Association
ALT	Alanine Aminotransferase
AST	Aspartate Aminotransferase
ATS	American Thoracic Society
BMI	Body mass index
CI	Confidence Interval
DLCO	Diffusing Capacity of the Lung for Carbon Monoxide
ERS	European Respiratory Society
FEV_1_	Forced Expiratory Volume in 1 s
fL	Femtoliter
FVC	Forced Vital Capacity
g/DL	Grams per Deciliter
g/L	Grams per Liter
GAP	Gender, age, physiology
GOR	Gastroozefagial reflux
HRCT	High-resolution computed tomography
JRS	Japanese Respiratory Society
IPF	Idiopathic pulmonary fibrosis
LDH	Lactate Dehydrogenase
LTOT	Long-term oxygen therapy
MDT-ILD	Multidisciplinary team-Interstitial lung disease
mg/DL	Milligrams per Deciliter
MPV	Mean Platelet Volume
NLR	Neutrophil-to-Lymphocyte Ratio
OR	Odds Ratio
PAH	Pulmonary arterial hypertension
PFT	Pulmonary function tests
RDW-CV	Red Cell Distribution Width–Coefficient of Variation
SD	Standard deviation
UIP	Usual interstitial pneumonia
U/L	Units per Liter
WBC	White Blood Cell count
×10^3^/μL	Thousand per microliter
